# Integrating Mercury Concentrations in American Alligators (*Alligator mississippiensis*) with Hunter Consumption Surveys to Estimate Exposure Risk

**DOI:** 10.1002/etc.5524

**Published:** 2023-01-13

**Authors:** Laura V. Kojima, Tracey D. Tuberville, Benjamin B. Parrott

**Affiliations:** ^1^ Savannah River Ecology Laboratory University of Georgia Aiken South Carolina USA; ^2^ Eugene P. Odum School of Ecology University of Georgia Athens Georgia USA; ^3^ Warnell School of Forestry and Natural Resources University of Georgia Athens Georgia USA

**Keywords:** Risk assessment, wildlife toxicology, metal accumulation, mercury, bioaccumulation

## Abstract

Mercury is a naturally occurring element but is also considered a widespread contaminant due to global anthropogenic activity. Even in moderate amounts, mercury (Hg) is an established neurotoxin and is associated with a range of adverse outcomes both in humans and wildlife. Humans in the United States are most commonly exposed to Hg through contaminated food or drinking water, and the consumption of game species, particularly those occupying higher trophic levels, has the potential to expose hunters to high concentrations of Hg. In the present study, we determined Hg concentrations in tail muscle and blood from American alligators (*Alligator mississippiensis*) inhabiting a region (Savannah River Site, SC, USA) with known Hg contamination. We then integrated these data with alligator harvest records and previously published surveys of alligator meat consumption patterns to estimate potential exposure risk. We found that the average Hg concentrations in tail muscle (1.34 mg/kg, wet wt) from sampled alligators exceeded the recommended threshold for Hg exposure based on the World Health Organization's guidelines (0.5 mg/kg, wet wt). In addition, based on regional consumption patterns reported for both adults and children, we estimated Hg exposures (${\bar{x}}_{\mathrm{Adult}}$ = 0.419 µg/kg/day, ${\bar{x}}_{\mathrm{Child}}$ = 2.24 µg/kg/day) occurring well above the US Environmental Protection Agency methylmercury reference dose of 0.1 μg/kg/day. Although the two reservoirs sampled in the present study are not currently open to alligator hunting, they are connected to waters that are publicly accessible, and the extent of alligator mobility across these sites is not known. Together, the findings reported in the present study further demonstrate the need for active monitoring of Hg concentrations in game species, which can convey substantial exposure risks to the public. *Environ Toxicol Chem* 2023;42:525–534. © 2023 The Authors. *Environmental Toxicology and Chemistry* published by Wiley Periodicals LLC on behalf of SETAC.

## INTRODUCTION

Although mercury (Hg) is a naturally occurring element, anthropogenic activity has resulted in global hotspots with elevated concentrations capable of exerting adverse health effects on wildlife and humans. For example, coal combustion, chlor‐alkali processing, and waste incineration introduce Hg into the environment through atmospheric transport and deposition (Driscoll et al., 2013; Jackson, 1997). This dynamic is particularly consequential in aquatic ecosystems harboring sulfate‐reducing bacteria, which convert Hg to its more toxic and bioavailable form, methylmercury (MeHg; Bank et al., 2005; Compeau & Bartha, 1985; Wagemann et al., 1997). Methylmercury bioaccumulates in organisms where it is readily absorbed into the bloodstream through the gastrointestinal tract and has a propensity to biomagnify across trophic levels (Bradley et al., 2017; Chumchal et al., 2011; Wolfe et al., 1998). Mercury has been documented to disrupt neuronal function in both humans and wildlife, negatively affecting coordination and movement, impairing both vision and speech, and weakening muscles (Sakamoto et al., 1998; US Environmental Protection Agency [USEPA], 2022). In addition, Hg has been found to negatively impact both the immune and endocrine systems, reproductive function, and in high amounts can result in mortality (Eisler, 2006; Scheuhammer et al., 2007; Tan et al., 2009; Todd et al., 2011; Wada et al., 2009; Wolfe et al., 1998).

In the United States, the main route of human Hg exposure is through direct ingestion from either contaminated food or water (Mahaffey, 2005; USEPA, 2022). The consumption of game species holds value across cultural, economic, and conservation contexts, but for certain species, consumption can also serve as a direct source of contaminant exposure, with attendant implications for public health (Arnett & Southwick, 2015; McCorquodale, 1997; Smith et al., 2018). In contrast to commercial agriculture, game species are often harvested from spatially complex and heterogeneous landscapes and occupy a range of different habitats and trophic positions. In aquatic systems, contaminant concentrations in fish populations are often monitored by state and federal governments, but other game species (such as waterfowl, ungulates, alligators, and rabbits) are typically not given the same level of attention (Conder & Arblaster, 2016; Smith et al., 2018). Globally, studies have investigated concentrations of contaminants in common game species, many of which have provided information on the risk of consuming game that has been killed with lead bullets and/or game meat that is sourced in close proximity to an area with known contamination (Arioli et al., 2019; Fachehoun et al., 2015; Morales et al., 2018; Oldenkamp et al., 2017; Swiergosz et al., 1993). While all game species can be exposed to contaminants, long‐lived predatory species living in contaminated environments have the potential to bioaccumulate high‐contaminant body burdens (Rowe, 2008), particularly for compounds such as Hg, which tend to biomagnify within an ecosystem.

The American alligator (*Alligator mississippiensis*) is an apex predator that inhabits a variety of freshwater and coastal habitats across 10 states in the southern United States, nine of which incorporate regulated alligator harvests into their wildlife management plans. Alligator harvests have served as an important mechanism for incentivizing the conservation of alligators and their habitat after these animals faced near extinction in the 1960s (Heykoop & Frechette, 2001). In addition to licensing fees that subsidize alligator management programs, direct and indirect economic benefits are realized for landowners, guides, and local communities (Louisiana Alligator Advisory Council, 2021; Powell, 2017). Due to their long lifespans (>60 years), high site fidelity, and high trophic status, alligators are an established bioindicator species for monitoring contaminants in aquatic environments (Lawson et al., 2020; Nifong & Silliman, 2017; Rosenblatt & Heithaus, 2011; Wilkinson et al., 2016). These same attributes also convey substantial exposure risks to individuals that consume their meat.

**Figure 1:**
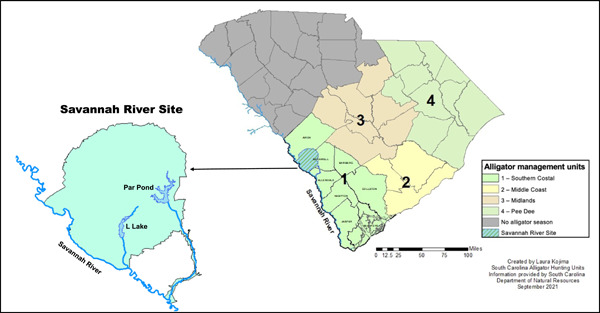
Map of South Carolina showing the location of alligator (*Alligator mississippiensis*) public hunt units and the Savannah River Site with respect to the Hunt Unit of concern (Unit 1—Southern Coastal). The map of the Savannah River Site denotes our sampling sites, Par Pond and L Lake, and their respective connections to the Savannah River.

The Savannah River forms the border between South Carolina and Georgia and is open to seasonal alligator harvests regulated by both states. Despite current consumption advisories for fish due to elevated Hg concentrations in the river, data on Hg concentrations in alligators are limited (South Carolina Department of Health and Environmental Control [SDHEC], 2020). Flanking the Savannah River, the Department of Energy's Savannah River Site is a former nuclear production plant which harbors reservoirs and wetlands with elevated concentrations of Hg (Jagoe et al., 1996; Figure 1). Although the Savannah River Site is not open to alligator hunting, it is possible that alligators move between the Savannah River Site and the Savannah River, making Hg‐exposed alligators on the Savannah River Site potentially available to hunters. In the present study, we quantified Hg concentrations in blood and tail muscle from alligators inhabiting the two former nuclear cooling reservoirs on the Savannah River Site that directly connect to the Savannah River through outfall streams. Our objectives were (1) to examine the relationship between body size and bioaccumulation of total Hg (THg) in harvest‐sized alligators, (2) to evaluate the relationship between THg concentrations in blood and tail muscle to determine if Hg concentrations in the former can serve as a proxy for concentrations in the latter, and (3) to use published hunter consumption data to assess potential THg exposure risk associated with consuming these alligators. We predicted that THg concentrations would increase with size in alligators, THg concentrations in blood would reflect those measured in tail muscle, and the consumption of alligators occupying the Savannah River Site would present significant Hg exposure risks to humans.

## METHODS

### Study area

The Department of Energy's Savannah River Site is a former nuclear production facility that houses two former nuclear reactor cooling reservoirs, Par Pond and L Lake. Elevated Hg concentrations in organisms inhabiting both reservoirs have been previously documented, likely originating from Hg‐contaminated water pumped from the Savannah River below a now inactive off‐site chlor‐alkali plant (Jagoe et al., 1996; Kvartek et al., 1994). Par Pond, the larger of the two reservoirs (1100 ha), was constructed in 1958 to dissipate heat effluent from the P and R reactors on site. In 1991, a precautionary drawdown and repair of the Par Pond dam resulted in the resuspension of contaminated sediment in the reservoir, causing an increase in bioavailable Hg (US Department of Energy, 1995). As a result, Hg concentrations in organisms inhabiting this reservoir have been well documented and monitored; however, Hg concentrations in alligators have not been assessed since the implementation of regulated harvests (Brisbin et al., 1992; Brown et al., 2022; Clay et al., 1978; Jagoe et al., 1998; Peles et al., 2006; Sugg et al., 1995; Yanochko et al., 1997). The smaller (405 ha) and newer of the two reservoirs, L Lake, was constructed in 1984 to dissipate heat effluent from the L Reactor. In contrast to Par Pond, the presence of Hg in L Lake biota has not received as much attention (Burger et al., 2003; Jagoe et al., 1996; Peles et al., 2006). Both reactors ceased operation in 1988 and their associated reservoirs currently serve as habitat for a variety of aquatic wildlife. Par Pond and L Lake drain into the Savannah River through two creeks, providing potential corridors for alligators to move between the reservoirs and the publicly accessible Savannah River (Figure 1).

### Alligator capture and sample collection

From July to August 2020 and May to July 2021, we captured alligators using baited trip‐snare traps (Murphy & Fendley, 1974), pole snares, or by hand. Immediately following capture, we collected blood samples from each alligator from the post‐occipital venous sinus with a sterile 20‐gauge needle and 30‐ml syringe, which were transferred to either a 3‐ or 8‐ml lithium heparin Vacutainer collection tube (BD) and kept on wet ice for no longer than 5 h before being frozen at −30 °C until analysis. We recorded morphological data including total length (cm) and sex, and gave unmarked individuals unique scute clip marks (Bustard & Choudhury, 1981; Jennings et al., 1991; Rainwater et al., 2007). In addition, we injected individuals subcutaneously with a passive integrated transponder tag (AVID) using a sterile 12‐gauge needle (Wilkinson et al., 2016) at the right lateral area near the proximal base of the tail. For individuals with a total length ≥180 cm, we collected tail muscle at the left lateral area near the proximal base of the tail using a 10‐mm Acuderm® biopsy punch (Acu‐Punch® by Acuderm). The South Carolina Department of Natural Resources permits harvesting alligators with a total length of ≥122 cm, but at least 95% of alligators harvested in South Carolina are ≥180 cm, thus we considered only alligators ≥180 cm to be harvestable size (Butfiloski, 2021; South Carolina Department of Natural Resources, 2019, 2020). To reduce potential discomfort, we administered 3 cc of 2% Lidocaine (Vet One®) to the biopsy site using a 5‐cc syringe and a 20‐gauge 1.5‐in needle prior to taking the biopsy. We released all alligators at their original capture location immediately following processing. We handled all alligators in accordance with approved protocols from the University of Georgia's Institutional Animal Care and Use Committee (AUP# A2020 02‐026‐Y2‐A0). Scientific collection permits for capturing, handling, and collecting samples from alligators were issued by the South Carolina Department of Natural Resources (permits #SC‐08‐2020 and #SC‐08‐2021).

### Quantifying THg in alligators

Prior to analysis, we thawed blood and tail muscle samples at room temperature. We homogenized blood samples using a vortex homogenizer for 30 s and placed a 50‐µl aliquot into a nickel weigh boat for analysis. We removed dermal tissue from the tail muscle biopsies using sterile shears and weighed each sample (wet wt to the nearest 0.001 g), freeze‐dried it, then homogenized it using a Wig‐L‐Bug® grinder (Wig‐L‐Bug® Amalgamator; Crescent Dental). Once prepared, we weighed 0.005 g of each muscle sample and transferred the sample to a nickel weigh boat for analysis. We quantified THg concentrations in blood and tail muscle (mg/kg, wet wt) using a Direct Mercury Analyzer (DMA‐80 EVO DUAL CELL, Milestone; hereafter DMA) at the Savannah River Ecology Laboratory, University of Georgia (Aiken, SC, USA). The DMA uses a combination of thermal decomposition, gold amalgamation, catalytic conversion, and atomic absorption spectrometry to determine the mass fraction of THg in solid or liquid samples. For quality control, each run of 10 samples incorporated a blank, a replicate, and a certified reference standard (TORT‐3; National Research Council of Canada). We originally obtained THg concentrations in tail muscle as dry weight (mg/kg), which we converted to wet weight (mg/kg) to account for sample preparation and phase differences (liquid vs. solid) between blood and tail muscle. We used the following formula to estimate the percentage of moisture content (*M*) for each tail muscle sample (Equation 1; Lawson et al., 2020; Lusk et al., 2005).

(1) $$M=\frac{{\text{TM}}_{W}{\;–\;\text{TM}}_{D}}{{\text{TM}}_{W}}\times 100$$

The tail muscle sample's THg dry weight estimate (dw) was then converted to wet weight (ww) using the derived percentage of moisture content (Equation 2).

(2) $$\text{ww}=\text{dw}\times \left(1–\frac{M}{100}\right)$$

T$M_{w}$ in Equation 1 refers to the total mass of the wet sample that we transferred to the cryovial prior to freeze‐drying, whereas T$M_{D}$ is the mass of the sample after freeze‐drying. All THg values are reported as mg/kg, wet weight.

### Statistical analysis

We performed all analyses with the statistical software RStudio v2021.09.1 (RStudio Team, 2021) and produced all figures using the package ggplot2 3.3.5 (Wickham, 2016). We tested data for normality and homogeneity of variance and log‐transformed when necessary to fit basic assumptions of analysis. Preliminary analysis performing a Student's *t*‐test found that THg in both tail muscle and whole blood did not differ significantly between sexes, therefore we did not consider sex in our models. We applied linear regression analyses to assess relationships between total length and THg concentrations in both tail and whole‐blood samples, in addition to evaluating the interaction between size and site on THg concentrations. A Student's *t*‐test was used to assess the relationship between tail muscle THg concentrations and site of capture, and whole‐blood THg concentrations and site of capture. Finally, we performed a Pearson correlation analysis to describe the relationship between blood THg and tail muscle THg values (both mg/kg, wet wt) based on individuals for which both sample types were available. The resulting regression constant and coefficient in Equation 3 were then used to estimated muscle THg concentration from whole‐blood THg measurements for those alligators (≥180 cm) for which a tail muscle sample was not collected.

(3) $$\begin{array}{l} y=a+bx\\ y=-.11+1.2x \end{array}$$

In Equation 3, *a* is the regression constant, which is the mean response variable when the predictor (THg blood) values are set at zero, whereas *b* is the regression coefficient. The *x* value in the equation is the predictor variable (THg blood concentrations) and *y* is the predicted tail muscle concentration (mg/kg, wet wt). We calculated descriptive statistics (mean, standard error, and range) of THg concentrations from tail muscle samples and their corresponding blood samples overall (Savannah River Site) and for each site separately (L Lake and Par Pond; Table 1).

**Table 1:** Total mercury (THg) concentrations in tail muscle and whole‐blood samples from American alligators (*Alligator mississippiensis*) from two sites on the Savannah River Site (SRS), South Carolina, USA

Site	Tail muscle (mg/kg, wet wt)	Whole blood (mg/kg, wet wt)	Predicted tail muscle (mg/kg, wet wt)
SRS	1.31 ± 0.18 (*n* = 31; 0.077–4.33)	0.938 ± 0.10 (*n* = 53; 0.076–3.41)	1.34 ± 0.15 (*n* = 40; 0.077–4.33)
Par Pond	1.97 ± 0.22 (*n* = 17; 0.453–4.33)	1.34 ± 0.18 (*n* = 24; 0.076–3.41)	1.97 ± 0.20 (*n* = 21; 0.453–4.33)
L Lake	0.510 ± 0.05 (*n* = 14; 0.077– 0.847)	0.602 ± 0.06 (*n* = 29; 0.095–1.21)	0.642 ± 0.08 (*n* = 19; 0.077–1.45)

Tail muscle was collected from individuals ≥180 cm total length and whole blood was collected from all captured individuals. Predicted tail muscle (*n* = 9 predicted) THg concentrations were estimated using the linear equation (Equation 3). Values are reported as mean ± SE (sample size; ranges).

#### Estimating consumption risk

We estimated consumption risk by combining our data on muscle THg concentrations in harvest‐sized alligators (≥180 cm total length) with published survey data of regional hunter consumption patterns of alligators (Tipton et al., 2017). Tipton et al. (2017) interviewed 23 recreational hunters who harvested alligators in South Carolina in 2015 and obtained information on planned consumption of the harvested meat, including the predicted meal size and predicted daily consumption frequency, to explore three consumption scenarios (lower bound, average, and upper bound scenarios). In addition, Tipton et al. (2017) acquired site‐specific information for two hunt units, including the Southern Coastal Unit (Figure 1), where the Savannah River Site resides. We applied these same consumption scenarios to our muscle THg data to determine potential daily exposure associated with consuming alligators collected on the Savannah River Site. In each scenario, adult (80 kg) and child (15 kg) body weights were used to account for exposure based on body weight. We examined dietary exposure scenarios for each reservoir and for both reservoirs combined to account for the risk of Hg exposure from alligators on one reservoir being higher than the other and to estimate the average risk of eating alligators that originate from the Savannah River Site. (Table 2).

**Table 2:** Values for three scenarios (lower bound, average, and upper bound) of harvested American alligator (*Alligator mississippiensis*) meat consumption using South Carolina survey data from Tipton et al. (2017) to determine the frequency of consumption (times/year) and amount per meal (oz)

Scenario	Frequency of consumption (times/year)	Amount/meal (oz)	THg concentration (mg/kg, wet wt)
Lower bound	2	3	0.077
Average	31	10.4	1.34
Upper bound	52	20.8	4.33

THg concentration (mg/kg; wet wt) values from Savannah River Site, South Carolina alligator tail muscle, including predicted values (*n* = 40; ≥180 cm total length).

THg = total mercury.

## RESULTS

### THg concentrations in alligator tissues

**Figure 2:**
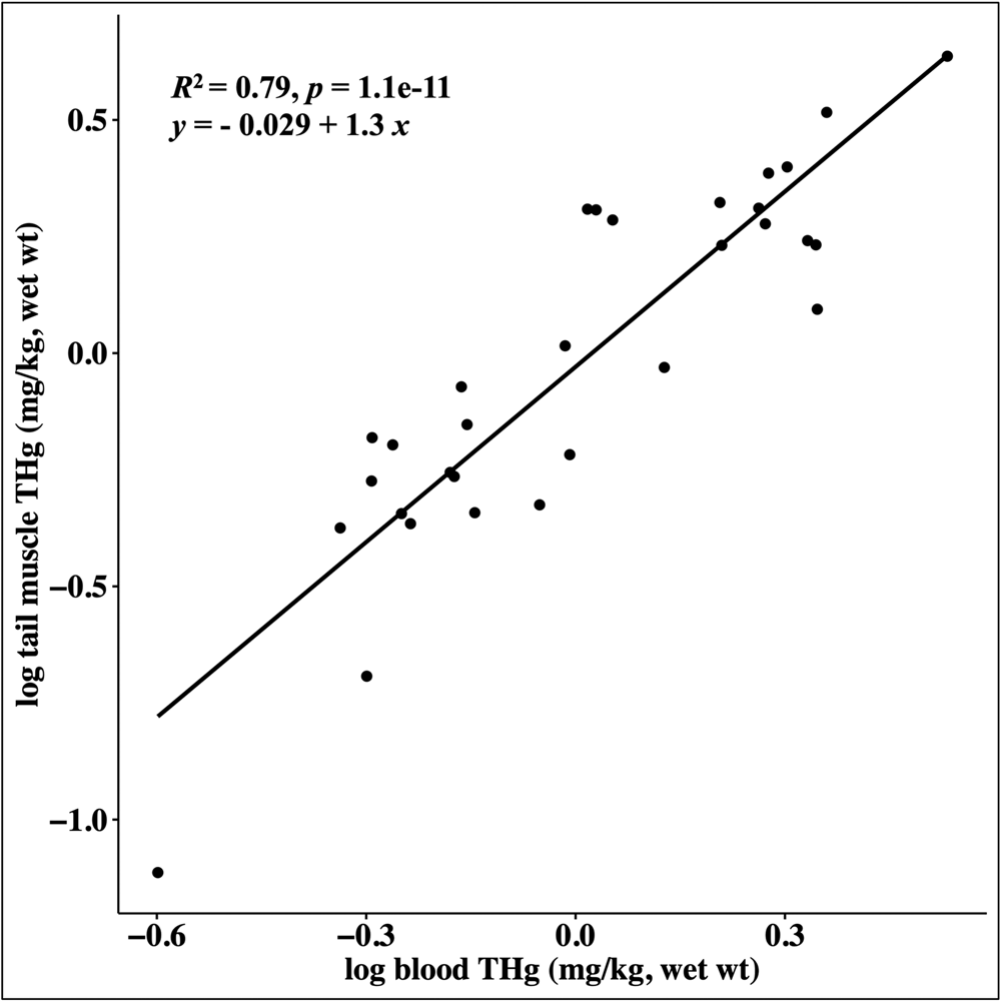
Pearson correlation (*R*) between blood and tail muscle total mercury (THg) concentrations in American alligators (*Alligator mississippiensis*) captured in Par Pond and L Lake on the Savannah River Site, South Carolina, USA (*n* = 31). All THg values are reported as wet weight.

**Figure 3:**
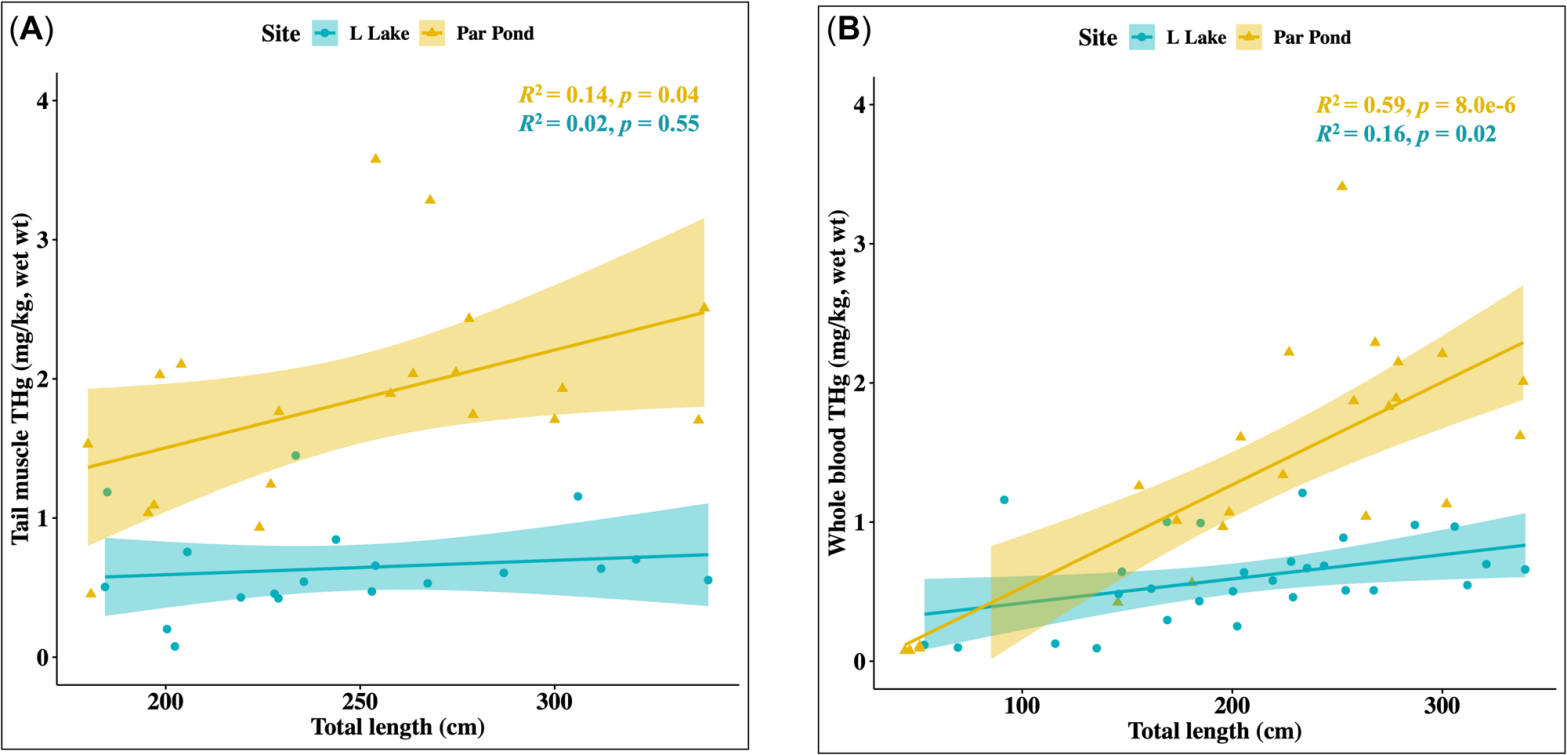
The relationship between total mercury (THg), total length, and site of capture in American alligators (*Alligator mississippiensis*) on the Savannah River Site. (**A**) Tail muscle THg was significantly correlated to total length in Par Pond, but not in L Lake. Tail muscle values were obtained from alligators ≥180 cm (*n* = 40). (**B**) Whole‐blood THg was significantly correlated to total length in both Par Pond and L Lake (*n = 53)*. Significant differences between THg concentrations in Par Pond and L Lake were observed, with a more notable trend of bioaccumulation in alligators on Par Pond.

**Figure 4:**
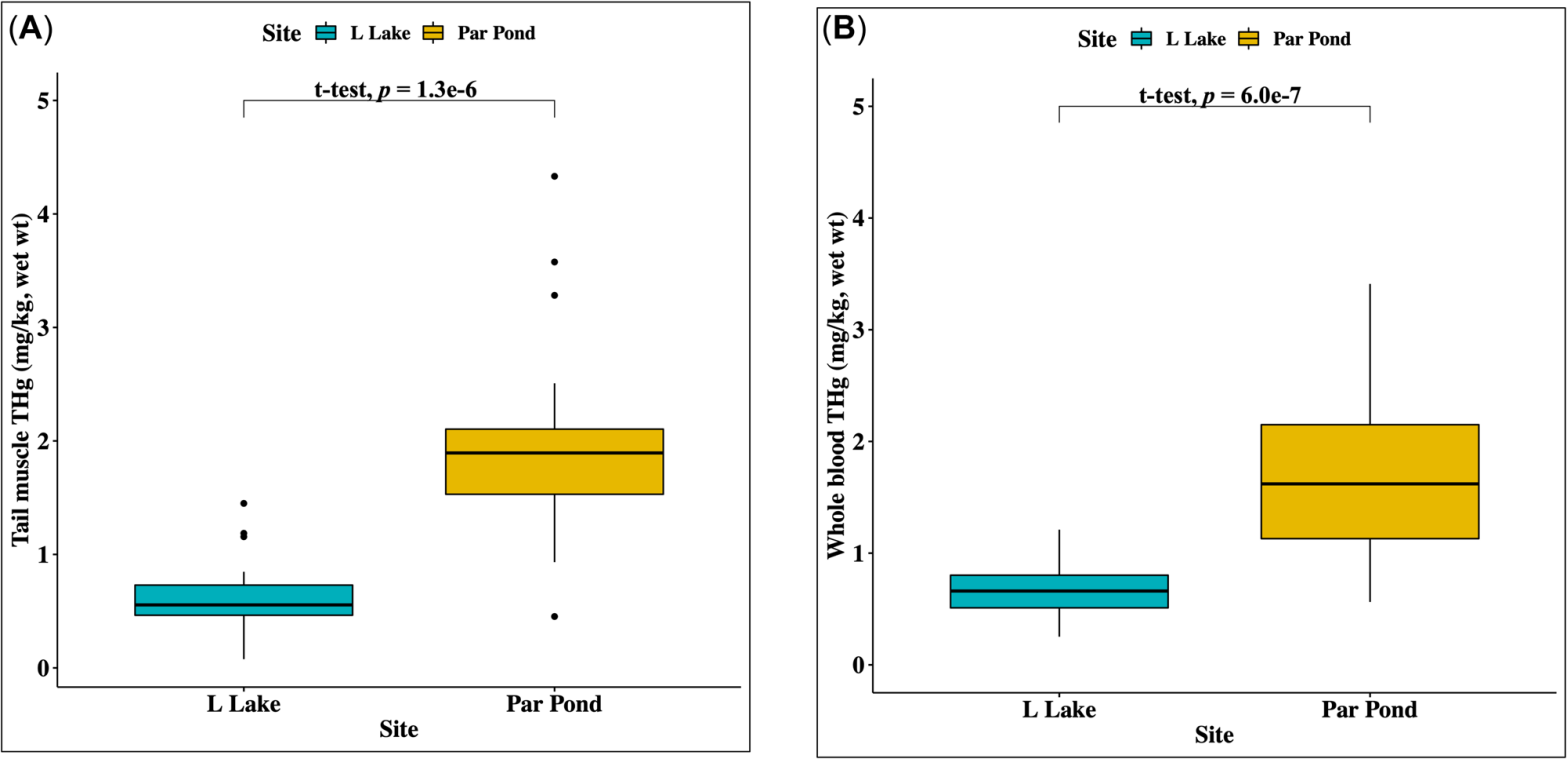
The relationship between tail and blood total mercury (THg) and site of capture in American alligators (*Alligator mississippiensis*) on the Savannah River Site. Boxplots represent tail and blood THg, including predicted values (*n* = 40). For both tail muscle and blood, alligators on Par Pond had significantly higher concentrations of THg: (**A**) *t* = −6.27, *p* = 1.3 × 10^−6^; (**B**) *t* = −6.60, *p* = 6.0 × 10^−7^. All THg values are reported as wet weight.

We sampled a total of 53 alligators across all size classes, 31 of which were harvestable alligators (total length ≥180 cm). Total Hg concentrations in Savannah River Site alligators ranged from 0.077 to 4.33 mg/kg in tail muscle (*n* = 31) and 0.076 to 3.41 mg/kg in whole blood (*n* = 53; Table 1). We found that blood and tail THg were significantly and positively correlated (*p* = 1.1 × 10^−11^, *R* ^2^ = 0.79, *n* = 31; Figure 2). Muscle THg increased with increasing total length; however, this relationship was not significant when considering data from both Par Pond and L Lake (*p* = 0.051, *R* ^2^ = 0.30, *n* = 40) nor was the interaction of total length and site (*p* = 0.17; Figure 3A). Whole‐blood THg concentrations and total length were positively correlated (*p* = 1.3 × 10^−5^, *R* ^2^ = 0.30, *n* = 53) and the interaction between total length and site was significant (*p* = 0.0004, *F* = 32.76; Figure 3B). In addition, harvest‐sized alligators (total length ≥180 cm) from Par Pond had significantly higher THg in tail muscle (x̄_*Par Pond*_ = 1.97 mg/kg, wet wt; x̄_*L Lake*_ = 0.64 mg/kg, wet wt; *p* = 1.3 × 10^–6^) and blood (x̄_*Par Pond*_ = 1.34 mg/kg, wet wt; x̄_*L Lake*_ = 0.60 mg/kg, wet wt; *p* = 6.0 × 10^−7^) when compared with harvest‐sized alligators from L Lake (Figure 4A,B).

### Potential consumption risk

Only alligators ≥180 cm were sampled for tail muscle and considered when analyzing consumption risk data. Additional tail muscle THg concentrations were estimated using the linear equation (Equation 3) for nine harvestable animals from which muscle samples were not collected (*n* = 40; 31 direct measurements, nine predicted). Mean THg concentration in tail muscle of harvest‐sized alligators from the Savannah River Site ($\bar{x}$ = 1.31 ± 0.18 mg/kg, wet wt) exceeded the World Health Organization's recommended consumption concentration (0.5 mg/kg, wet wt; Ikem & Egiebor, 2005). When examining different exposure scenarios based on consumption rates and mealsize survey data, there was a wide range of predicted exposures (summarized in Table 3). Potential dietary exposure across the Savannah River Site ranged from 0.036 to 363.92 µg/day with an average exposure of 33.54 µg/day. Estimated daily dietary exposure for adults ranged from 0.00045 to 4.55 µg/kg body weight/day, and using the average consumption scenario (average frequency × average serving size × average Hg concentration) we found the average dietary exposure for an adult was 0.419 µg/kg body weight per day. In the case of children, estimated daily dietary exposure ranged from 0.002 to 7.23 µg/kg body weight/day. This wide range in predicted THg exposure among scenarios can be attributed to the differences in THg concentrations in alligators between reservoirs, with the highest concentrations coming from Par Pond.

**Table 3:** Potential daily dietary exposure of mercury (Hg; µg/day) from harvested alligator (*Alligator mississippiensis*) meat using South Carolina alligator harvest survey data from Tipton et al. (2017) and tail muscle Hg concentrations from alligators (≥180 cm total length) sampled on the Savannah River Site

		Total (µg/day)	Adult (µg/kg body wt per day)	Child (µg/kg body wt per day)
*SRS* (*n* = 40)				
Lower bound	Low frequency × small serving × low conc.	0.036	0.00045	0.002
Upper bound	High frequency × large serving × high conc.	363.92	4.55	24.26
Average	Avg. frequency × avg. serving × avg. conc.	33.54	0.419	2.24
*Par Pond* (*n* = 21)				
Lower bound	Low frequency × small serving × low conc.	0.211	0.003	0.014
Upper bound	High frequency × large serving × high conc.	363.92	4.55	24.26
Average	Avg. frequency × avg. serving × avg. conc.	49.34	0.612	3.29
*L‐Lake* (*n* = 19)				
Lower bound	Low frequency × small serving × low conc.	0.036	0.00045	0.002
Upper bound	High frequency × large serving × high conc.	121.79	1.52	8.12
Average	Avg. frequency × avg. serving × avg. conc.	16.07	0.201	1.07

SRS = Savannah River Site; avg. = average; conc. = concentration.

## DISCUSSION

### THg concentrations in alligator whole blood and tail muscle

Our findings are consistent with Hg being prevalent in aquatic environments throughout the southeastern United States, where consumption of game species harvested through recreational hunting can lead to significant dietary exposures. Prior studies have used blood as a minimally invasive sample to evaluate Hg concentrations in crocodilians (Burger et al., 2007; Eggins et al., 2015; Lawson et al., 2020; Lemaire et al., 2021; Nilsen et al., 2017), and our findings that whole‐blood THg concentrations can be used to infer tail muscle THg concentrations are consistent with those studies. For example, Nilsen et al. (2017) observed a similar relationship between concentrations of THg in the blood and muscle of alligators in Florida, and reported that blood concentrations of selenium, rubidium, and zinc are also reflective of those in tail muscle. Strong correlations between THg in blood and other tissues have also been documented in alligators from other study sites and in other reptiles, such as brown watersnakes (*Nerodia taxispilota*), collected on the Savannah River (Haskins, Brown, Bringolf, et al., 2021; Haskins, Brown, Qin, et al., 2021; Moore et al., 2022). Blood sampling protocols are usually less invasive than biopsy procedures and downstream analytical protocols are typically less time‐consuming. Taken together with findings from other studies, our results suggest that whole blood represents a minimally invasive and reliable proxy for THg concentrations in alligator tail muscle, which could be used in future monitoring applications.

We found that THg concentrations in blood were positively correlated with alligator length, which is in contrast to previous studies at the Savannah River Site reporting only scute Hg concentrations were correlated with alligator length (Jagoe et al., 1998; Yanochko et al., 1997). Studies at the Tom Yawkey Wildlife Center in South Carolina and Merritt Island National Wildlife Refuge in Florida have observed a nonlinear relationship between whole‐blood THg and alligator length, suggesting accumulation occurs until growth cessation (Lawson et al., 2020). In black caimans (*Melanosuchus niger*) in French Guiana, a linear relationship between whole‐blood THg and total length was documented (Lemaire et al., 2021), and correlations between whole‐blood THg and total length have been observed in other reptiles, although the results are not always consistent (Burger et al., 2007; Eggins et al., 2015; Schneider et al., 2011). The relationship between tail muscle THg and total length in our study was not significant when considering combined data from the reservoirs. When examining this relationship separately at each reservoir, we found that the relationship was significant on Par Pond, but not on L Lake; however, analyses of tail muscle were restricted to samples from larger individuals (≥180 cm) and thus lacked the variation in length included in our analyses of blood. Whereas there is broad support for the bioaccumulation of Hg across diverse taxa, increases in THg with crocodilian length might not be solely attributable to bioaccumulation dynamics alone and can reflect ontogenetic shifts in diet (Platt et al., 2006, 2013; Wallace & Leslie, 2008). Crocodilians, including alligators, typically feed at lower trophic levels during juvenile life stages compared with adults (Nifong et al., 2015). For example, in black caimans, THg in whole blood was positively correlated with both trophic position and total length (Lemaire et al., 2021). Similarly, THg liver and muscle concentrations in alligators in Texas were positively correlated with trophic position (Chumchal et al., 2011). Additional studies aimed at parsing the relative contributions of bioaccumulation and biomagnification via ontogenetic dietary shifts are promising avenues for better resolving the ecotoxicological dynamics that drive THg body burdens in crocodilians.

### Consumption risk

Our results suggest the consumption of alligator tail muscle has the potential to convey substantial dietary exposure to Hg. The World Health Organization recommends not consuming food with Hg levels ≥0.5 mg/kg, wet weight and the average THg concentrations in tail muscle from alligators on the Savannah River Site were well above this limit (Ikem & Egiebor, 2005). In addition, the US Food and Drug Administration (USFDA) recommends a limit of 0.46 mg/kg, wet weight for weekly human consumption, but frequent consumption of food products with Hg that have low concentrations (e.g., three servings per week that are ≤0.15 mg/kg, wet wt) are permissible (USFDA, 2021). We derived daily dietary exposure of Hg from alligator tail muscle from lower and upper bound scenarios of consumption as well as the average consumption scenario. In addition, we considered how exposure varies between adults and children and calculated the daily exposure for each group (Table 3). We showed that exposure can vary depending on the site from which the alligators originated, with consumption of alligators from Par Pond resulting in greater exposure compared with L Lake. However, because the reservoir of origin for alligators harvested off the Savannah River Site is likely to be unknown, we also estimated exposure for the Savannah River Site as a whole by combining values from the two reservoirs. Data from Tipton et al. (2017) showed that 45.5% of surveyed hunters reported consuming 3 oz of harvested alligator meat in a sitting, with 63.6% reporting an expected consumption of alligator meat greater than or equal to once a month. Given the elevated Hg concentrations observed in alligators inhabiting the Savannah River Site, hunters that consume alligator meat from this area put themselves at a high risk of Hg exposure if they do not limit their consumption to at most one serving (3 oz) once a month. Other factors that influence THg exposure include sharing of harvested meat with children and vulnerable groups within a household, sharing with other households, and consumption of other game species (Smith et al., 2018). Tipton et al. (2017) reported that more than 45% of successful alligator hunters in South Carolina planned to share alligator meat with children under 15 years old, with half of these individuals disclosing that their children were likely to consume alligator meat at the same frequency they did (Tipton et al., 2017). Along with pregnant women, children are at greatest risk of the health effects of Hg exposure (USFDA, 2021). Children are more vulnerable to high Hg exposure due to the negative effects on their developing systems (Bose‐O'Reilly et al., 2010; Sly & Pronczuk, 2007; WHO, 2006). Thus, there are several factors to be considered when predicting risk of Hg exposure from alligator consumption, including animal body burdens and site of origin, as well as consumption dynamics (e.g., frequency of consumption, body wt, portion size). In addition, overall Hg exposure risk will be influenced by the extent to which hunters consume other game species—an aspect that has received little attention (Smith et al., 2018).

Our analysis did not differentiate what proportion of THg is composed of MeHg. However, muscle THg concentrations of brown watersnakes (*Nerodia taxispilota*) on the Savannah River are strongly correlated with MeHg, with the highest average percentage of MeHg in THg being found in muscle (79.4% ± 1.7%; Haskins, Brown, Bringolf, et al., 2021). Similarly, MeHg comprises an average of 90% of the THg in fish muscle (Burger et al., 2014; Mason et al., 2000). In addition, Chumchal et al. (2011) documented that in alligators in Texas, MeHg in muscle was highly correlated with THg (81.6%), and in a study considering two crocodile species (*Melanosuchus niger* and *Caiman crocodilus*) Gomes et al. (2020) found that MeHg in muscle tissues comprised between 84% and 94% of THg. Based on existing evidence, it is likely that MeHg is the primary contributor to THg measured in alligator tail muscle in the present study and thus exceeds the reference dose (MeHg = 0.1 µg/kg/day) for most consumption scenarios (USFDA, 2021).

Currently, there is no direct evidence of alligators moving between the Savannah River Site and the adjacent Savannah River. However, alligators have been documented to frequently move between water bodies, some more than 10 km (Fujisaki et al., 2014; Rosenblatt & Heithaus, 2011; Subalusky et al. 2009). During the course of our study, we captured two alligators on L Lake that were initially captured on Par Pond (~7.7 km apart) in 2002 and 2007. The distance from L Lake to the Savannah River is approximately 8 km, suggesting that alligator movement from the Savannah River Site to the Savannah River is possible, if not likely. The Savannah River itself has a history of Hg contamination, probably related to a chlor‐alkali plant upstream of the Savannah River Site that was active in the 1970s and nearby coal ash basins on the Savannah River Site (Kvartek et al., 1994). Due to elevated Hg concentrations in the Savannah River, the SDHEC has issued consumption advisories for common fish species in the river, such as largemouth bass (*Micropterus salmoides*), chain pickerel (*Esox niger*), and spotted suckers (*Minytrema melanops*; SDHEC, 2020). Other fish species, such as bowfin (*Amia calva*) and largemouth bass downstream of the Savannah River Site, are prohibited from being fished due to elevated Hg concentrations (Burger et al., 2001; SDHEC, 2020). Because many of these fish species are likely to contribute to the alligator diet, consumption advisories for alligators from the Savannah River may also be warranted. Localized sampling of alligators (such as on the Savannah River) and an understanding of their landscape‐scale movement patterns would help tailor consumption advisories to appropriately consider localized patterns of risk associated with alligator consumption.

## Disclaimer

This report was prepared as an account of work sponsored by an agency of the United States government. Neither the United States government nor any agency thereof, nor any of their employees, makes any warranty, express or implied, or assumes any legal liability or responsibility for the accuracy, completeness, or usefulness of any information, apparatus, product, or process disclosed. Reference herein to any specific commercial product, process, or service by trade name, trademark, or manufacturer, or otherwise does not necessarily constitute or imply its endorsement, recommendation, or favoring by the United States government or any agency thereof. The view and opinions of authors expressed herein do not necessarily state or reflect those of the United States government or any agency thereof.

## Author Contributions Statement

**Laura Kojima**: Conceptualization; Data curation; Formal analysis; Funding acquisition; Investigation; Methodology; Project administration; Validation; Visualization; Writing—original draft; Writing—review & editing. **Tracey Tuberville**: Conceptualization; Funding acquisition; Investigation; Methodology; Project administration; Resources; Supervision; Validation; Writing—original draft; Writing—review & editing. **Benjamin B. Parrott**: Conceptualization; Funding acquisition; Investigation; Methodology; Project administration; Resources; Supervision; Validation; Writing—original draft; Writing—review and editing.

## Data Availability

We have additional publications in the works using these data and will release data on request. All data, including raw data and calculation tools, are available on request from the corresponding author (laura.kojima@uga.edu).

## References

[etc5524-bib-0001] Arioli, F. , Ceriani, F. , Nobile, M. , Vigano, R. , Besozzi, M. , Panseri, S. , & Chiesa, L. M. (2019). Presence of organic halogenated compounds, organophosphorus insecticides and polycyclic aromatic hydrocarbons in meat of different game animal species from an Italian subalpine area. Food Additives and Contaminants A, 36(8), 1244–1252. 10.1080/19440049.2019.1627003 31192775

[etc5524-bib-0002] Arnett, E. B. , & Southwick, R. (2015). Economic and social benefits of hunting in North America. International Journal of Environmental Studies, 72(5), 734–745. 10.1080/00207233.2015.1033944

[etc5524-bib-0003] Bank, M. S. , Loftin, C. S. , & Jung, R. E. (2005). Mercury bioaccumulation in northern two‐lined salamanders from streams in the northeastern United States. Ecotoxicology, 14(1), 181–191. 10.1007/s10646-004-6268-8 15931966

[etc5524-bib-0004] Bose‐O'Reilly, S. , McCarty, K. M. , Steckling, N. , & Lettmeier, B. (2010). Mercury exposure and children's health. Current Problems in Pediatric and Adolescent Health Care, 40(8), 186–215. 10.1016/j.cppeds.2010.07.002 20816346 PMC3096006

[etc5524-bib-0005] Bradley, M. A. , Barst, B. D. , & Basu, N. (2017). A review of mercury bioavailability in humans and fish. International Journal of Environmental Research and Public Health, 14(2), 169. 10.3390/ijerph14020169 28208586 PMC5334723

[etc5524-bib-0006] Brisbin, I. L. , Benner, J. M. , Brandt, L. A. , Kennamer, R. A. , & Murphy, T. M. (1992). *Long‐term population studies of American alligators inhabiting a reservoir: Initial responses to water level drawdown*. Proceedings of the Eleventh Working Meeting. IUCN/SSC Crocodile Specialist Group, The World Conservation Union (pp. 53–76).

[etc5524-bib-0007] Brown, M. K. , Haskins, D. L. , Russell, A. L. , Lambert, M. L. , Quick, C. E. , Pilgrim, M. A. , & Tuberville, T. D. (2022). Mercury and radiocesium accumulation and associations with sublethal endpoints in the Florida green watersnake (*Nerodia floridana*). Environmental Toxicology and Chemistry, 41(3), 758–770. 10.1002/etc.5281 35112731

[etc5524-bib-0008] Burger, J. , Campbell, K. R. , Murray, S. , Campbell, T. S. , Gaines, K. F. , Jeitner, C. , Shukla, T. , Burke, S. , & Gochfeld, M. (2007). Metal levels in blood, muscle and liver of water snakes (*Nerodia* spp.) from New Jersey, Tennessee and South Carolina. Science of the Total Environment, 373(2), 556–563. 10.1016/j.scitotenv.2006.06.018 17239425

[etc5524-bib-0009] Burger, J. , Dixon, C. , Boring, S. , & Gochfeld, M. (2003). Effect of deep‐frying fish on risk from mercury. Journal of Toxicology and Environmental Health, Part A, 66(9), 817–828. 10.1080/15287390306382 12746129

[etc5524-bib-0010] Burger, J. , Gaines, K. F. , & Gochfeld, M. (2001). Ethnic differences in risk from mercury among Savannah River fishermen. Risk Analysis, 21, 533–544. 10.1111/0272-4332.213130 11572431

[etc5524-bib-0011] Burger, J. , Gochfeld, M. , Jeitner, C. , Donio, M. , & Pittfield, T. (2014). Sushi consumption rates and mercury levels in sushi: Ethnic and demographic differences in exposure. Journal of Risk Research, 17(8), 981–997. 10.1080/13669877.2013.822925

[etc5524-bib-0013] Butfiloski, J. (2021). Private lands alligator hunting guide. South Carolina Department of Natural Resources. https://dc.statelibrary.sc.gov/bitstream/handle/10827/31273/DNR_SC_Private_Lands_Alligator_Program_2019-2020.pdf?sequence=1%26isAllowed=y

[etc5524-bib-0014] Chumchal, M. M. , Rainwater, T. R. , Osborn, S. C. , Roberts, A. P. , Abel, M. T. , Cobb, G. P. , Smith, P. N. , & Bailey, F. C. (2011). Mercury speciation and biomagnification in the food web of Caddo Lake, Texas and Louisiana, USA, a subtropical freshwater ecosystem. Environmental Toxicology and Chemistry, 30(5), 1153–1162. 10.1002/etc.477 21305578

[etc5524-bib-0015] Clay, D. L. , Brisbin, I. L., Jr., P. B. Bush , & Provost, E. E. (1978). Patterns of mercury contamination in a wintering waterfowl community. Proceedings of the Annual Conference of the Southeastern Association of Fish and Wildlife Agencies, 32, 309–317.

[etc5524-bib-0016] Compeau, G. C. , & Bartha, R. (1985). Sulfate‐reducing bacteria: Principal methylators of mercury in anoxic estuarine sediment. Applied and Environmental Microbiology, 50(2), 498–502. 10.1128/aem.50.2.498-502.1985 16346866 PMC238649

[etc5524-bib-0017] Conder, J. M. , & Arblaster, J. A. (2016). Development and use of wild game consumption rates in human health risk assessments. Human and Ecological Risk Assessment: An International Journal, 22(1), 251–264. 10.1080/10807039.2015.1060407

[etc5524-bib-0018] Driscoll, C. T. , Mason, R. P. , Chan, H. M. , Jacob, D. J. , & Pirrone, N. (2013). Mercury as a global pollutant: Sources, pathways, and effects. Environmental Science & Technology, 47(10), 4967–4983. 10.1021/es305071v 23590191 PMC3701261

[etc5524-bib-0019] Eggins, S. , Schneider, L. , Krikowa, F. , Vogt, R. C. , Da Silveira, R. , & Maher, W. (2015). Mercury concentrations in different tissues of turtle and caiman species from the Rio Purus, Amazonas, Brazil. Environmental Toxicology and Chemistry, 34(12), 2771–2781. 10.1002/etc.3151 26387493

[etc5524-bib-0020] Eisler, R. (2006). Mercury hazards to living organisms (1st ed.). CRC Press.

[etc5524-bib-0021] Fachehoun, R. C. , Lévesque, B. , Dumas, P. , St‐Louis, A. , Dubé, M. , & Ayotte, P. (2015). Lead exposure through consumption of big game meat in Quebec, Canada: Risk assessment and perception. Food Additives & Contaminants. Part A, Chemistry, 32(9), 1501–1511. 10.1080/19440049.2015.1071921 26161681

[etc5524-bib-0022] Fujisaki, I. , Hart, K. M. , Mazzotti, F. J. , Cherkiss, M. S. , Sartain, A. R. , Jeffery, B. M. , Beauchamp, J. S. , & Denton, M. (2014). Home range and movements of American alligators (*Alligator mississippiensis*) in an estuary habitat. Animal Biotelemetry, 2(1), 8. 10.1186/2050-3385-2-8

[etc5524-bib-0023] Gomes, D. F. , Moreira, R. A. , Sanches, N. A. O. , do Vale, C. A. , Daam, M. A. , Gorni, G. R. , & Bastos, W. R. (2020). Dynamics of (total and methyl) mercury in sediment, fish, and crocodiles in an Amazonian Lake and risk assessment of fish consumption to the local population. Environmental Monitoring and Assessment, 192(2), 101. 10.1007/s10661-020-8066-z 31916004

[etc5524-bib-0024] Haskins, D. L. , Brown, M. K. , Bringolf, R. B. , & Tuberville, T. D. (2021). Brown watersnakes (*Nerodia taxispilota*) as bioindicators of mercury contamination in a riverine system. Science of the Total Environment, 755, 142545. 10.1016/j.scitotenv.2020.142545 33038814

[etc5524-bib-0025] Haskins, D. L. , Brown, M. K. , Qin, C. , Xu, X. , Pilgrim, M. A. , & Tuberville, T. D. (2021). Multi‐decadal trends in mercury and methylmercury concentrations in the brown watersnake (*Nerodia taxispilota*). Environmental Pollution, 276, 116722. 10.1016/j.envpol.2021.116722 33640654

[etc5524-bib-0026] Heykoop, J. , & Frechette, D. L. (2001). Gatornomics: Profitable and sustainable use of alligators in the southeastern United States. Marine Resource Economics, 16(2), 127–142. http://www.jstor.org/stable/42628834

[etc5524-bib-0027] Ikem, A. , & Egiebor, N. (2005). Assessment of trace elements in canned fishes (Mackerel, Tuna, Salmon, Sardines and Herrings) marketed in Georgia and Alabama (United States of America). Journal of Food Composition and Analysis, 18, 771–787. 10.1016/j.jfca.2004.11.002

[etc5524-bib-0028] Jackson, T. A. (1997). Erratum: Long‐range atmospheric transport of mercury to ecosystems, and the importance of anthropogenic emissions-a critical review and evaluation of the published evidence. Environmental Reviews, 5(3–4), 207. 10.1139/a97-012

[etc5524-bib-0029] Jagoe, C. H. , Shaw‐Allen, P. L. , & Brundage, S. (1996). Gill Na+, K+ ‐ATPase activity in largemouth bass (*Micropterus salmoides*) from three reservoirs with different levels of mercury contamination. Aquatic Toxicology, 36(3), 161–176. 10.1016/S0166-445X(96)00814-4

[etc5524-bib-0030] Jagoe, C. H. , Arnold‐Hill, B. , Yanochko, G. M. , Winger, P. V. , & Brisbin, I. L., Jr. (1998). Mercury in alligators (*Alligator mississippiensis*) in the southeastern United States. Science of the Total Environment, 213(1), 255–262. 10.1016/S0048-9697(98)00098-9 9652131

[etc5524-bib-0031] Jennings, M. L. , David, D. N. , & Portier, K. M. (1991). Effect of marking techniques on growth and survivorship of hatchling alligators. Wildlife Society Bulletin, 19(2), 204–207. http://www.jstor.org/stable/3782331

[etc5524-bib-0032] Kvartek, E. J. , Carlton, W. H. , Denham, M. , Eldridge, L. , & Newman, M. C. (1994). *Assessment of mercury in the Savannah River Site environment* (WSRC‐TR‐94‐0218. Technical report for the US Department of Energy under contract No. DE‐AC09‐89SR18035). US Department of Energy. 10.2172/263920

[etc5524-bib-0033] Lawson, A. J. , Moore, C. T. , Rainwater, T. R. , Nilsen, F. M. , Wilkinson, P. M. , Lowers, R. H. , Guillette, L. J. , McFadden, K. W. , & Jodice, P. G. R. (2020). Nonlinear patterns in mercury bioaccumulation in American alligators are a function of predicted age. Science of the Total Environment, 707, 135103. 10.1016/j.scitotenv.2019.135103 31863991

[etc5524-bib-0034] Lemaire, J. , Bustamante, P. , Marquis, O. , Caut, S. , & Brischoux, F. (2021). Influence of sex, size and trophic level on blood Hg concentrations in Black caiman, *Melanosuchus niger* (Spix, 1825) in French Guiana. Chemosphere, 262, 127819. 10.1016/j.chemosphere.2020.127819 32768753

[etc5524-bib-0035] Louisiana Alligator Advisory Council . (2021). *Louisiana Alligator Program management*. https://www.louisianaalligators.com/alligator-management-program.html

[etc5524-bib-0036] Lusk, J. D. , Rich, E. , & Bristol, R. S. (2005). Methylmercury and other environmental contaminants in water and fish collected from four recreational fishing lakes on the Navajo Nation, 2004. US Fish and Wildlife Service, Albuquerque, New Mexico, USA.

[etc5524-bib-0037] Mahaffey, K. R. (2005). Mercury exposure: Medical and public health issues. Transactions of the American Clinical and Climatological Association, 116, 127–154. 16555611 PMC1473138

[etc5524-bib-0038] Mason, R. P. , Laporte, J.‐M. , & Andres, S. (2000). Factors controlling the bioaccumulation of mercury, methylmercury, arsenic, selenium, and cadmium by freshwater invertebrates and fish. Archives of Environmental Contamination and Toxicology, 38(3), 283–297. 10.1007/s002449910038 10667925

[etc5524-bib-0039] McCorquodale, S. M. (1997). Cultural contexts of recreational hunting and native subsistence and ceremonial hunting: Their significance for wildlife management. Wildlife Society Bulletin, 25(2), 568–573. http://www.jstor.org/stable/3783493

[etc5524-bib-0040] Moore, L. A. , Finger, J. W. , Haskins, D. L. , Elsey, R. M. , Castleberry, S. B. , Glenn, T. C. , Jagoe, C. H. , & Brisbin, I. L., Jr. (2022). Tissue distribution of mercury in the bodies of wild American alligators (*Alligator mississippiensis*) from a coastal marsh in Louisiana (USA). Archives of Environmental Contamination and Toxicology, 83(1), 13–20. 10.1007/s00244-022-00938-3 35699748

[etc5524-bib-0041] Morales, J. S. , Moreno‐Ortega, A. , Amaro Lopez, M. A. , Arenas Casas, A. , Cámara‐Martos, F. , & Moreno‐Rojas, R. (2018). Game meat consumption by hunters and their relatives: A probabilistic approach. Food Additives & Contaminants, Part A: Chemistry, Analysis, Control, Exposure & Risk Assessment, 35(9), 1739–1748. 10.1080/19440049.2018.1488183 29912678

[etc5524-bib-0042] Murphy, T. M. , & Fendley, T. T. (1974). A new technique for live trapping of nuisance alligators. Proceedings of the Annual Conference of the Southeastern Association Of Game and Fish Commissioners, 27, 308–311.

[etc5524-bib-0043] Nifong, J. C. , Layman, C. A. , & Silliman, B. R. (2015). Size, sex and individual‐level behaviour drive intrapopulation variation in cross‐ecosystem foraging of a top‐predator. Journal of Animal Ecology, 84(1), 35–48. 10.1111/1365-2656.12306 25327480

[etc5524-bib-0044] Nifong, J. C. , & Silliman, B. (2017). Abiotic factors influence the dynamics of marine habitat use by a highly mobile “freshwater” top predator. Hydrobiologia, 802(1), 155–174. 10.1007/s10750-017-3255-7

[etc5524-bib-0045] Nilsen, F. M. , Kassim, B. L. , Delaney, J. P. , Lange, T. R. , Brunell, A. M. , Guillette, L. J. , Long, S. E. , & Schock, T. B. (2017). Trace element biodistribution in the American alligator (*Alligator mississippiensis*). Chemosphere, 181, 343–351. 10.1016/j.chemosphere.2017.04.102 28456036 PMC11314667

[etc5524-bib-0046] Oldenkamp, R. E. , Bryan, A. L. , Kennamer, R. A. , Leaphart, J. C. , Webster, S. C. , & Beasley, J. C. (2017). Trace elements and radiocesium in game species near contaminated sites. The Journal of Wildlife Management, 81(8), 1338–1350. https://www.jstor.org/stable/26608506

[etc5524-bib-0047] Peles, J. D. , Glenn, T. C. , Brant, H. A. , Wall, A. K. , & Jagoe, C. H. (2006). Mercury concentrations in largemouth bass (*Micropterus salmoides*) from five South Carolina Reservoirs. Water, Air, and Soil Pollution, 173(1), 151–162. 10.1007/s11270-005-9034-5

[etc5524-bib-0048] Platt, S. , Rainwater, T. , Finger, A. , Thorbjarnarson, J. , Anderson, T. , & McMurry, S. (2006). Food habits, ontogenetic dietary partitioning and observations of foraging behaviour of Morelet's crocodile (*Crocodylus moreletii*) in northern Belize. The Herpetological Journal, 16, 281–290.

[etc5524-bib-0049] Platt, S. , Thorbjarnarson, J. , Rainwater, T. , & Martin, D. (2013). Diet of the American Crocodile (*Crocodylus acutus*) in marine environments of coastal Belize. Journal of Herpetology, 47, 1–10. 10.1670/12-077

[etc5524-bib-0050] Powell, J. K. (2017). Alligator harvesting: Hunting as a regulatory tool for species management. The Florida Bar Journal, 91(2), 22.

[etc5524-bib-0051] RStudio Team . (2021). *RStudio: Integrated Development Environment for R*. RStudio, PBC. http://www.rstudio.com/

[etc5524-bib-0052] Rainwater, T. R. , Wu, T. H. , Finger, A. G. , Cañas, J. E. , Yu, L. , Reynolds, K. D. , Coimbatore, G. , Barr, B. , Platt, S. G. , Cobb, G. P. , Anderson, T. A. , & McMurry, S. T. (2007). Metals and organochlorine pesticides in caudal scutes of crocodiles from Belize and Costa Rica. The Science of the Total Environment, 373(1), 146–156. 10.1016/j.scitotenv.2006.11.010 17182086

[etc5524-bib-0053] Rosenblatt, A. E. , & Heithaus, M. R. (2011). Does variation in movement tactics and trophic interactions among American alligators create habitat linkages? Journal of Animal Ecology, 80(4), 786–798. 10.1111/j.1365-2656.2011.01830.x 21418209

[etc5524-bib-0054] Rowe, C. L. (2008). “The calamity of so long life”: Life histories, contaminants, and potential emerging threats to long‐lived vertebrates. BioScience, 58(7), 623–631. 10.1641/B580709

[etc5524-bib-0055] Sakamoto, M. , Wakabayashi, K. , Kakita, A. , Hitoshi, T. akahashi , Adachi, T. , & Nakano, A. (1998). Widespread neuronal degeneration in rats following oral administration of methylmercury during the postnatal developing phase: A model of fetal‐type Minamata disease. Brain Research, 784(1), 351–354. 10.1016/S0006-8993(97)01400-5 9518689

[etc5524-bib-0056] Scheuhammer, A. M. , Meyer, M. W. , Sandheinrich, M. B. , & Murray, M. W. (2007). Effects of environmental methylmercury on the health of wild Birds, mammals, and fish. AMBIO: A Journal of the Human Environment, 36(1), 12–19. 10.1579/0044-7447(2007)36[12:EOEMOT]2.0.CO;2 17408187

[etc5524-bib-0057] Schneider, L. , Belger, L. , Burger, J. , Vogt, R. C. , Jeitner, C. , & Peleja, J. R. P. (2011). Assessment of non‐invasive techniques for monitoring mercury concentrations in species of Amazon turtles. Toxicological & Environmental Chemistry, 93(2), 238–250. 10.1080/02772248.2010.517627

[etc5524-bib-0058] Sly, P. D. , & Pronczuk, J. (2007). Guest editorial: Susceptibility of children to pollutants. Paediatric Respiratory Reviews, 8(4), 273–274. 10.1016/j.prrv.2007.07.011 18005893

[etc5524-bib-0059] Smith, J. B. , Tuberville, T. D. , & Beasley, J. C. (2018). Hunting and game consumption patterns of hunters in South Carolina. Journal of Fish and Wildlife Management, 9(1), 321–329. 10.3996/032017-JFWM-028

[etc5524-bib-0060] South Carolina Department of Health and Environmental Control . (2020). *Current South Carolina fish consumption advisories*. https://scdhec.gov/sites/default/files/media/document/Advisory%20Table%20March%202020.pdf

[etc5524-bib-0061] South Carolina Department of Natural Resources . (2019). *South Carolina Department of Natural Resources 2019 alligator harvest data*.

[etc5524-bib-0062] South Carolina Department of Natural Resources . (2020). *South Carolina Department of Natural Resources 2020 alligator harvest data*.

[etc5524-bib-0063] Subalusky, A. L. , Fitzgerald, L. A. , & Smith, L. L. (2009). Ontogenetic niche shifts in the American Alligator establish functional connectivity between aquatic systems. Biological Conservation, 142(7), 1507–1514. 10.1016/j.biocon.2009.02.019

[etc5524-bib-0064] Sugg, D. W. , Chesser, R. K. , Brooks, J. A. , & Grasman, B. T. (1995). The association of DNA damage to concentrations of mercury and radiocesium in largemouth bass. Environmental Toxicology & Chemistry, 14(4), 661. 10.1002/etc.5620140414

[etc5524-bib-0065] Swiergosz, R. , Perzanowski, K. , Makosz, U. , & Biłek, I. (1993). The incidence of heavy metals and other toxic elements in big game tissues. The Science of the Total Environment, Suppl Pt, 1, 225–231. 10.1016/s0048-9697(05)80022-1 8108705

[etc5524-bib-0066] Tan, S. W. , Meiller, J. C. , & Mahaffey, K. R. (2009). The endocrine effects of mercury in humans and wildlife. Critical Reviews in Toxicology, 39(3), 228–269. 10.1080/10408440802233259 19280433

[etc5524-bib-0067] Tipton, J. J. , Guillette, L. J. , Lovelace, S. , Parrott, B. B. , Rainwater, T. R. , & Reiner, J. L. (2017). Analysis of PFAAs in American alligators part 2: Potential dietary exposure of South Carolina hunters from recreationally harvested alligator meat. Journal of Environmental Sciences, 61, 31–38. 10.1016/j.jes.2017.05.046 PMC6526952 29191313

[etc5524-bib-0068] Todd, B. D. , Bergeron, C. M. , Hepner, M. J. , Burke, J. N. , & Hopkins, W. A. (2011). Does maternal exposure to an environmental stressor affect offspring response to predators. Oecologia, 166(1), 283–290. 10.1007/s00442-011-1961-9 21416404 PMC3074075

[etc5524-bib-0069] US Department of Energy . (1995). *Envrionmental assessment for the natural fluctuation of water level in Par Pond and reduced water flow in Steel Creek below L‐Lake at the Savannah River Site*. Report No. DOE/EA‐1070. https://www.energy.gov/nepa/downloads/ea-1070-revised-finding-no-significant-impact

[etc5524-bib-0070] US Environmental Protection Agency . (2022). *Health effects of exposures to mercury*. https://www.epa.gov/mercury/health-effects-exposures-mercury

[etc5524-bib-0072] US Food and Drug Administration . (2021). *Technical information on development of FDA/EPA advice on eating fish*. Center for Food Safety and Applied Nutrition. https://www.fda.gov/food/metals-and-your-food/technical-information-development-fdaepa-advice-about-eating-fish-those-who-might-become-or-are

[etc5524-bib-0073] Wada, H. , Cristol, D. A. , McNabb, F. M. A. , & Hopkins, W. A. (2009). Suppressed adrenocortical responses and thyroid hormone levels in birds near a mercury‐contaminated river. Environmental Science & Technology, 43(15), 6031–6038. 10.1021/es803707f 19731714

[etc5524-bib-0074] Wagemann, R. , Trebacz, E. , Hunt, R. , & Boila, G. (1997). Percent methylmercury and organic mercury in tissues of marine mammals and fish using different experimental and calculation methods. Environmental Toxicology and Chemistry, 16(9), 1859–1866. 10.1002/etc.5620160914

[etc5524-bib-0075] Wallace, K. M. , & Leslie, A. J. (2008). Diet of the Nile Crocodile (*Crocodylus niloticus*) in the Okavango Delta, Botswana. Journal of Herpetology, 42(2), 361–368. http://www.jstor.org/stable/40060522

[etc5524-bib-0076] Wickham, H. (2016). ggplot2: Elegant graphics for data analysis. Springer‐Verlag.

[etc5524-bib-0077] Wilkinson, P. M. , Rainwater, T. R. , Woodward, A. R. , Leone, E. H. , & Carter, C. (2016). Determinate growth and reproductive lifespan in the American alligator (*Alligator mississippiensis*): Evidence from long‐term recaptures. Copeia, 104(4), 843–852. 10.1643/CH-16-430

[etc5524-bib-0078] Wolfe, M. F. , Schwarzbach, S. , & Sulaiman, R. A. (1998). Effects of mercury on wildlife: A comprehensive review. Environmental Toxicology and Chemistry, 17(2), 146–160. 10.1002/etc.5620170203

[etc5524-bib-0079] World Health Organization (WHO) . (2006). Principles for evaluating health risks in children associated with exposure to chemicals‐environmental health criteria (Vol. 237, pp. 1–329).

[etc5524-bib-1074] Yanochko, G. M. , Jagoe, C. H. , & Brisbin Jr., I. L. , (1997). Tissue mercury concentrations in alligators (Alligator mississippiensis) from the Florida Everglades and the Savannah River Site, South Carolina. Archives of Environmental Contamination and Toxicology, 32(3), 323–328. 10.1007/s002449900192 9096083

